# Analysis Polyadenylation Signal Usage in *Sus scrofa*

**DOI:** 10.3390/ani12020194

**Published:** 2022-01-13

**Authors:** Yuting Zhang, Jingwen Song, Min Zhang, Zhongyuan Deng

**Affiliations:** 1 School of Agricultural Sciences, Zhengzhou University, Zhengzhou 450001, China; zhyuting2021@163.com (Y.Z.); songjingwen0704@foxmail.com (M.Z.); 2 School of Life Sciences, Zhengzhou University, Zhengzhou 450001, China; zhangmin753@gmail.com

**Keywords:** *Sus scrofa*, polyadenylation, polyadenylation signal, poly(A), APA

## Abstract

**Simple Summary:**

RNA polyadenylation is an important step of eukaryotic gene expression and the progress depends on a highly conserved AAUAAA hexamer motif, known as the polyadenylation signal (PAS). We identified polyadenylation signals in *Sus scrofa*, the PAS motif similar with other mammalians. APA (alternative polyadenylation) analysis show that most gene was affect by the alternative polyadenlation in *Sus scrofa*. The PAS data presented in this manuscript will be useful for *Sus scrofa* research by facilitating the improved annotation of the *Sus scrofa* genome and post-transcriptionally regulation mode.

**Abstract:**

RNA polyadenylation is an important step in the messenger RNA (mRNA) maturation process, and the first step is recognizing the polyadenylation signal (PAS). The PAS type and distribution is a key determinant of post-transcriptional mRNA modification and gene expression. However, little is known about PAS usage and alternative polyadenylation (APA) regulation in livestock species. Recently, sequencing technology has enabled the generation of a large amount of sequencing data revealing variation in poly(A) signals and APA regulation in *Sus scrofa*. We identified 62,491 polyadenylation signals in *Sus scrofa* using expressed sequence tag (EST) sequences combined with RNA-seq analysis. The composition and usage frequency of polyadenylation signal in *Sus scrofa* is similar with that of human and mouse. The most highly conserved polyadenylation signals are AAUAAA and AUUAAA, used for over 63.35% of genes. In addition, we also analyzed the U/GU-rich downstream sequence (DSE) element, located downstream of the cleavage site. Our results indicate that APA regulation was widely occurred in *Sus scrofa*, as in other organisms. Our result was useful for the accurate annotation of RNA 3′ ends in *Sus scrofa* and the analysis of polyadenylation signal usage in *Sus scrofa* would give the new insights into the mechanisms of transcriptional regulation.

## 1. Introduction

RNA polyadenylation, addition of a 3′ poly(A) tail at the cleavage site (CS) of a pre-mRNA, is an important step of eukaryotic gene expression, affecting mRNA stability, translation, transport, and other post-transcriptional processes [1,2,3,4,5,6]. The polyadenylation process is regulated by two core *cis*-acting elements, various accessary *cis*-acting elements and diverse *trans*-acting protein factors including the polyadenylation machinery and RNA binding proteins (RBPs). The two core *cis*-acting elements are a highly conserved AAUAAA hexamer motif, known as the polyadenylation signal (PAS), 10–30 bp 5′ to the cleavage site, and a more variable U/UG-rich element 15–30 bp 3′ of the cleavage site [7]. The PAS is the binding site for the cleavage and polyadenylation specificity factor (CPSF) [8,9], one protein complex of the polyadenylation machinery. The polyadenylation machinery is composed of four subcomplexes: (1) cleavage and polyadenylation specificity factor (CPSF); (2) cleavage stimulation factor (CSTF); (3) cleavage factor I (CFIm); and (4) cleavage factor II (CFIIm). Working as a complex, CPSF recognizes the polyadenylation signal sequence AAUAAA and cleaves the pre-mRNA [10].

The downstream U/UG-rich element is the binding site of the cleavage-stimulating factor (CstF) [11], another key polyadenylation machinery protein complex. In silico analysis of the poly (A) tail-containing transcripts from human, mouse, freshwater planarian (*Schmidtea mediterrane*) and fruit fly (*Drosophila melanogaster*) reveals that the canonical PAS, AAUAAA, is present only in 40–49% of the mRNAs, 25~40% of the mRNAs contain a single-nucleotide variant of the canonical AAUAAA hexamer motif, and the remaining 13–25% of the mRNAs have no recognizable AAUAAA-like hexamer motif [11,12,13,14,15,16,17,18]. AAGAAA is another PAS hexamer with low frequency (~3%) which is often found as the PAS in an upstream exon, while the AAUAAA hexamer is most often associated with the last PAS in human and mouse pre-mRNAs [19].

Research found that universal and conservative AAUAAA varied widely among six eukaryotic species (yeast, rice, Arabidopsis, fruit flies, mice and humans). In animals, the highly conserved polyadenylation signals are AAUAAA and its one- or two-nucleotide variants (e.g., AUUAAA) located in the near upstream elements region [20]. Mutations in PAS and other poly(A) cis-elements, and the resulting alteration in gene expression have been shown to cause several human Mendelian diseases, such as IPEX syndrome and α-Thalassaemia [21,22]. Given the high prevalence of APA, there are likely many more common SNPs that modulate APA and affect disease susceptibility. PAS SNPs potentially have a strong impact on cleavage and polyadenylation efficiency at their downstream pA (poly(A)) sites. Each PAS SNP divides its 3′UTR into two segments: a common UTR (cUTR) which is upstream to the associated pA site and is common to the transcript generated by cleavage at this pA site and the transcripts generated by usage of more distal pA sites, and an alternative UTR (aUTR) which is included only in transcripts generated by more distal pA sites [23]. The effects of point mutations in AAUAAA hexamer have been determined, which showed that there are some disease-associated variants in PAS. For example, the NAA10 polyadenylation signal variants cause syndromic microphthalmia [24]. The emerging evidences indicate that polyadenylation signal could be potentially a biomarker for disease diagnosis, and possibly a novel therapy target in the future. Gene editing targeting the polyadenylation signal offers us another way of therapy. Facioscapulohumeral dystrophy (FSHD) is the most common dystrophy in adults and so far, there is no treatment. Different loci of the disease have been characterized and they all lead to the aberrant expression of the DUX4 protein, which impairs the function of the muscle, ultimately leading to cell death. Some experiments were conducted using gene editing to try to permanently shut down DUX4 expression by targeting its poly(A) sequence. The results suggest that targeting the DUX4 poly(A) signal to destroy the PAS is possible to some extent [25], hence a more effective method needs further studies. Single nucleotide exchanges within the hexamer are known to interfere tremendously with mRNA processing and cause a range of diseases. Small aptamer-based regulatory devices can be designed to control a range of RNA-dependent cellular processes and emerged as promising tools for fine-tuning gene expression in synthetic biology. Research achieved a polyadenylation-modulating riboswitch with a modest dynamic range which can be functional with different poly(A) signals and proved the modularity of the switch by exchanging the sensor module to a tetracycline aptamer [26].

Many genes have multiple polyadenylation (pA) sites and undergo alternative polyadenylation (APA) to form several different mRNA isoforms with variable length of 3′ coding sequence (coding sequence APA) and/or 3′ untranslated regions (3′UTR; UTR APA) [7,13,16,27,28,29,30,31]. The coding sequence APA can yield two or more alternative proteins with different C-terminal sequences and possibly different functions. The UTR APA can alter the stability of the mRNA and its ability to be bound by RNA-binding proteins or microRNA even though the protein sequence is unchanged [9,32]. The *cis*-acting elements specifying the multiple pA sites of a given gene are an important determinant of which pA site becomes used, and the selection of which pA site is used determines how that gene is regulated.

The raising of the domestic pig (*Sus scrofa*)*,* a major livestock animal farmed for meat production. While the pig genome has been sequenced [33], little is known about the 3′-processing of its 21,640 annotated protein-coding genes. By mapping 1.92 million polyadenylated (starting or ending with at least 10 As or Ts) RNA-seq reads onto the *Sus scrofa* genome, Wang [34] identified a total of 28,363 pA sites, 47% (13,033) and 53% (15,330) of which were located in 7403 annotated protein coding genes and non-annotated gene regions, respectively. Among the 7403 genes with identified pA sites, 41% of which harboured alternative pA sites [34]. However, the core *cis*-acting elements that specify these pig pA sites remain unknown. In this study, we identified a total of 62,491 pA sites by mapping 105,103 expressed sequence tags (ESTs) and 352,764,416 reads with a poly(A) tail onto the *Sus scrofa* genome. We further analyzed the nucleotide and motif distribution/frequency within 100 bp up- and down-stream of each of the 62,491 pA sites to identify the core *cis*-acting elements defining these pA sites. We found that 50.96% of the 62,491 pig pA sites contained the conserved AAUAAA hexamer, which was greater than that of human (46.82%) and mouse (39.54%) [17]. We also uncovered that 10.64% of the pig pA sites had no recognizable AAUAAA-like hexamer, which was lower than that of human (13%) and mouse (25%) [17]. These results indicate that the patterns of variant polyadenylation signal usage in the pig.

## 2. Materials and Methods

### 2.1. The Source of the EST Data and RNA Sequence Data

Sanger-based EST sequences were collected from The National Center for Biotechnology Information (NCBI) GenBank (http://www.ncbi.nlm.nih.gov/genbank/, accessed date: 31 July 2018). RNA-seq data were collected by searching for *Sus scrofa* and downloaded from the NCBI Sequence Read Archive database (http://www.ncbi.nlm.nih.gov/sra/, accessed date: 31 July 2018). The detailed information of the SRA accession number is provided in Supplementary Table S1. Fastq-dump is a tool of the NCBI SRA Toolkit, and was used to dump and extract SRA data into fastq format.

### 2.2. RNA Sequence Data Trimming

To avoid the various errors in the high-throughput sequencing data, a tool of the FASTX-Toolkit (http://hannonlab.cshl.edu/fastx_toolkit/download.html (accessed on 31 July 2018), the version: 0.0.14), the FASTQ Quality Filter, was used to remove low quantity reads. Next, FASTQ/A Clipper was used to remove sequence adapters if present.

### 2.3. Identification of Poly(A) Sites in the EST and RNA-Seq Data

**Figure 1:**
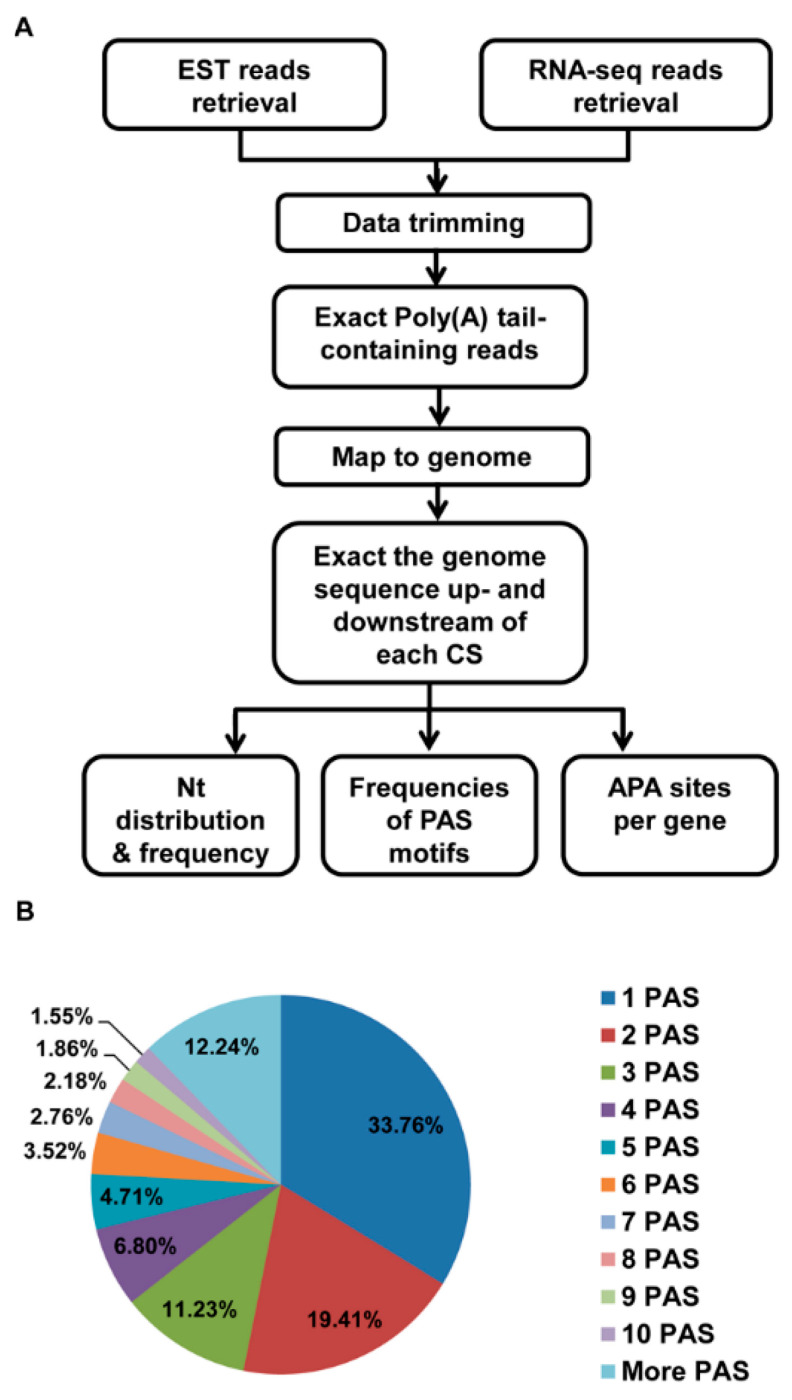
Identification of poly(A) sites and pA clusters. (**A**) The outline of using the RNA-seq and EST data to extract sequences of poly(A) sites and PAS in *Sus scrofa*. The RNA-seq data were obtained from the transcriptomes of *Sus scrofa* and EST data were obtained from the NCBI EST database. The data were first trimmed and the useless data were removed by filtering. The remaining data were mapped to the genome. The sequence upstream and downstream of the mapped reads and the tags were then extracted, and then the false sites from genome inter poly(A) regions were removed. The final data were used to analyze the PAS. (**B**) PASs considered as a cluster by separating 20 or less nt.

As shown in Figure 1, we identified putative novel polyadenylation sites. To do this, we first identified all sequencing reads that either began or ended with a run of adenine (A) or thymine (T) and were at least 10 nt in length, similar to the criteria used by Tian [13]. We then trimmed off the run of As or Ts and extracted the 50 bp of sequence before the polyadenylation sites. Then, all EST data and RNA-seq data were mapped to the genome using bowtie2. For the reads that mapped uniquely to the genome, we used samtools to infer the precise base where cleavage occurred. To filter out apparent cleavage sites that were potentially due to sequencing errors, we removed putative polyadenylation sites where the downstream genomic regions contained at least eight As or Ts, reasoning that a sequencing error at the non-A or T site might allow mis-mapping. Finally, we extracted the sequence 100 bp upstream and downstream for analysis.

### 2.4. Polyadenylation Signal Search and Analysis

We used the 100 bp upstream and 100 bp downstream of the cleavage site to analyze single-nucleotide and dual-nucleotide profiles. Then, we analyzed all 6 hexamer motifs from 0–50 bp, including the motif position and the number of genes that use this motif. Then, we selected 14 motifs and analyzed the AATAAA 1 bp variation frequency in the 40 bp upstream of the poly(A) site. Next, we analyzed the PAS distribution and analyzed the downstream U/GU richness.

### 2.5. APA Analysis

To analyze the presence of alternative polyadenyaltion in *Sus scrofa*, we downloaded the *Sus scrofa* gene annotation information (http://asia.ensembl.org/Sus_scrofa/Info/Index (accessed on 31 July 2018), the version: Sscrofa10.2.84). However, this annotation remains incomplete. We used 2 kb as the PAS position, and the presence of more than one PAS sequence suggested the potential for alternative polyadenylation. We analyzed the number of poly(A) sites in each gene.

### 2.6. PAS Annotation

We extracted the mapped information for the poly(A) tail reads and obtained the PAS locations in the chromosome. We then used the genome annotation file to extract the gene number in the different chromosomes and we analyzed the PAS location in the 5′UTR, CDS, and 3′UTR or intron according to this annotation.

### 2.7. Gene Ontology (GO) and Kyoto Encyclopedia of Genes and Genomes (KEGG) Pathway Enrichment Analysis

To understand the higher-level functions of the identified APA genes with more than two PASs, we performed GO term annotation and KEGG pathway enrichment analysis using the OmicShare tools, a free online platform for data analysis (https://www.omicshare.com/tools). APA genes were determined by GO terms of biological processes, cellular components and molecular functions. The statistical enrichment of selected genes in KEGG pathways with *p* values < 0.05 were considered significantly enriched.

## 3. Results

### 3.1. Identification of pA Sites

As described in Figure 1A, we retrieved all the publicly available raw EST and RNA-seq reads of *Sus scrofa* from the NCBI SRA database (https://www.ncbi.nlm.nih.gov/sra (accessed on 31 July 2018)). These included a total of 1,676,405 ESTs and 36,890,214,409 RNA-seq raw reads (Table 1). After removing low quantity reads and the reads not starting or ending with at least 10 As or Ts, we obtained a total of 105,103 EST sequences and 352,764,416 RNA-seq reads with a poly(A) tail. We then mapped these poly(A) tail-containing reads to the *Sus scrofa* genome (susScr3 and SGSC Sscrofa10.2 (NCBI project 13421, GCA_000003025.4, WGS AEMK01); The URL link: http://hgdownload.soe.ucsc.edu/goldenPath/susScr3/bigZips) (accessed on 31 July 2018). In addition, we got a total of 11,008,425 uniquely-mapped cleavage sites (69,602 from the 105,103 EST reads and 10,938,823 from 352,764,416 RNA-seq reads). By removing internally primed cleavage sites (The downstream of the cleavage site contained at least eight consecutive A in the genome) and merging closely spaced cleavage sites (two or more site separated less than or 20 bp), we identified a total of 62,491 pA sites (14,845 from the 69,602 EST reads and 60,277 from 10,938,823 RNA-seq reads) (Table 1). In this case, 33.76% of them had only one cleavage site, showing no heterogeneity (Figure 1B). The remaining pA sites contained two (19.41%) or more (46.83%) alternative cleavage sites (Figure 1B).

### 3.2. Nucleotide Bias near the Cleavage Sites

**Figure 2:**
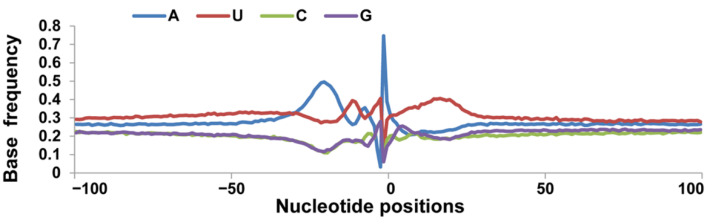
Distribution frequencies of A, C, G and U within 100 bp of the cleavage site of the poly(A) tail-containing transcripts of *Sus scrofa*. Single nucleotide profiles around the poly(A) sites. The *Y*-axis was used to plot the frequency of single nucleotides (A/U/G/C). Sequence upstream of the poly(A) site was from the −100 to 0 region and sequence downstream of the poly(A) site was from 0 to +100. The CS is considered position 0.

To identify the core *cis*-acting elements that define the 62,491 pA sites identified above, we analyzed the mononucleotide profiles of the 62,491 pA sites within 100 bp up- and down-stream of their cleavage sites. By chance, A, C, G and U should have a frequency of 25% at every position in this region. The mononucleotide profiles (Figure 2), however, showed that U frequency remained >25%, except for at position 0 (the cleavage site), at which its frequency was about 11.54%. U also had 3 frequency peaks, located at position 4–26, −5 to 0, and −8 to −12, respectively. The 4–26 U peak corresponded to the downstream core U/UG-rich elements specifically recognized by CstF. “A” frequency stayed around 25%, except for the 3 peaks at position 0, −28 to −15, and −8 to −6 as well as 1 trough at position 4–26 where U was overrepresented. Position −28 to −15 represented an A-rich region correspond the core AAUAAA such as hexamer element recognized by CPSF. The A peak at position 0 (frequency 74.63%) corresponded to the cleavage site recognized by the CFI and CFII complex. By contrast, C and G frequency remained below 25%, except for at position −1 (both C and G) and 4–5 (G only) where they reached 25% or slightly higher (Figure 2). The G peak at 4–5 and the U peak at 4–26 constituted the downstream U/GU-rich element. 

**Figure 3:**
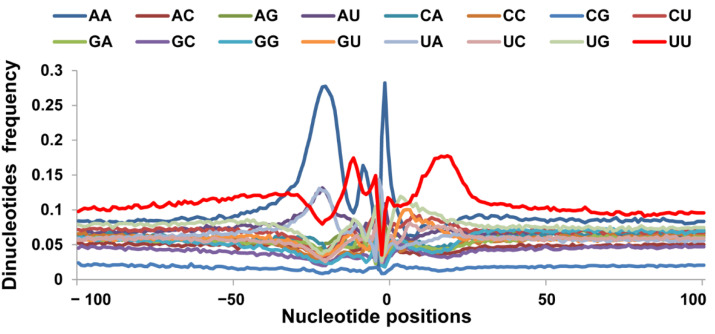
Distribution frequency of the dinucleotides within the 100 bp of the cleavage site. Dinucleotide profiles downstream of the poly(A) sites were determined to explore U/GU richness. The *Y*-axis represents the ratio of nucleotide number to total PAS number. Downstream of the poly(A) site was from −100 to 100 relative to the CS at position 0.

Since CA and UU/GU are enriched at the cleavage site (position −1 and 0) and 15–30 bp 3′ of the cleavage site, respectively, in many eukaryotes, we also examined the dinucleotide (AA, AC, AG, AU, CA, CC, CG, CU, GA, GC, GG, GU, UA, UC, UG, UU) profiles of the 62,491 pA sites within 100 bp up- and down-stream of their cleavage sites (Figure 3). The AA rich has a similar tendency with the single A rich in the Figure 3, AA was also overrepresented from position 30 to 100, with a frequency of >0.08, AC, AG, AU no obvious trend. CA has a sharp in the region −1 to 1, which was proved that the CA was cleavage site. The CC, CG and CU did not own this situation. Compared to the CA, the GA has a similar tendency with the CA except the small proportion. The UA has sharp on the region −1 to 1 and also own a little wide peaks at the region of 70 to 82 (Figure 3). The UU was highly enriched from −3 to −18 and 7 to 27 region, especially position 7 to 27, which corresponded to the downstream U/GU-rich element. The other dinucleotides occurred more or less randomly from position 1 to 100, with a frequency of 0.04 to 0.08 (Figure 3). 

### 3.3. Sequence Motifs near the Cleavage Sites

**Figure 4:**
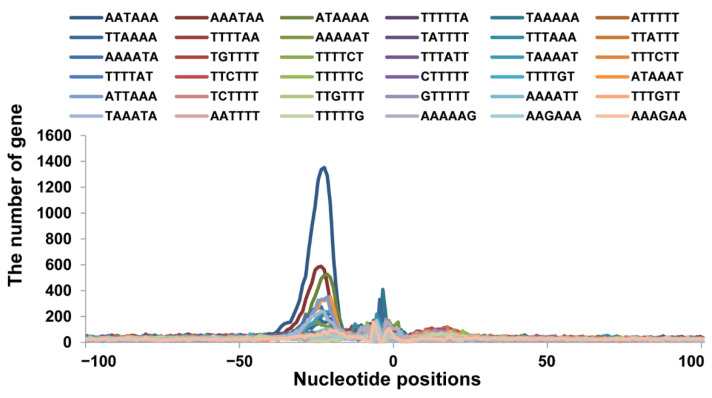
Distribution and number of random hexamers within the 100 bp of the cleavage site of the poly(A) tail-containing reads. Hexamer profiles upstream of the poly(A) sites to explore the candidate PAS sequences. The *Y*-axis represents the number of genes. Upstream of the poly(A) site was from −100 to 100 relative to the CS at position 0.

To find out the types of polyadenylation signals used in the pig, we scanned the 100 bp up or downstream of the cleavage sites of the 62,491 pA sites identified above for all the possible 6-base hexamers (Figure 4). A scattered distribution of a hexamer of low frequency would suggest a randomly occurring noise, whereas a peaked distribution of a hexamer of high frequency would be a true hexamer signal. By these criteria, AAUAAA exhibited the highest sharp peak between the −10 and −25 nt, suggesting it is the most common polyadenylation signal. The single-nucleotide variant AUUAAA, ACUAAA, and AGUAAA also exhibited sharp peaks between the −10 and −25 nt. The other variants such as UAUAAA, AAUAUA, AAUACA, CAUAAA, GAUAAA and AAUAGA also showed peaks between the −10 and −25 nt locations, which indicated that these hexamers are also PAS. However, the single-nucleotide variants AAGAAA and ACUAAA showed peaks located at −18 nt and −19 nt, respectively, although the peaks were broad. The variants AAAUAA and AUAAAA also have a peak upstream of the cleavage site, but may be influenced by the AAUAAA, as part of this sequence is shared. The variant UUUUUU did not exhibit a pronounced peak and showed a scattered distribution, which mean it was not a true PAS.

### 3.4. Usage Frequencies of AAUAAA and Single Nucleotide Variants Polyadenylation Signal Variants

Out of the 62,491 identified poly(A) sites, 89.36% contained at least one PAS hexamer in the 40 nt upstream of poly(A) sites (Table 2). Table 2 shows that the frequency of the canonical AAUAAA was 50.69%, higher than those reported previously for human and mouse. The AUUAAA polyadenylation signals were the second most frequently used hexamers (12.39%). None of the 14 PAS were found in the remaining 10.64% of pA sites, a lower fraction than that observed for human and mouse.

### 3.5. Distribution of the Identified pA Sites in the Pig Genome

**Figure 5:**
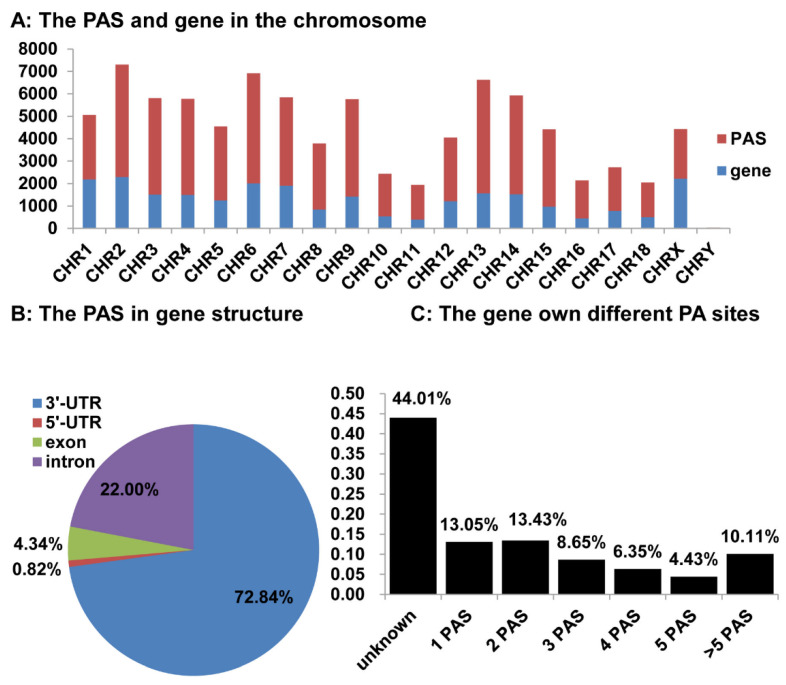
The alternative polyadenylation in *Sus scrofa*. (**A**) The number of PAS sites and their location was in the chromosome. We analyzed the PAS number distribution in the chromosome. The blue represents the gene number in the chromosome and the red represents the PAS number. (**B**) The PAS location in the gene structure. We analyzed the PAS occurrence frequency in the 5′UTR, exon, intron, and 3′UTR of genes. (**C**) The number of *Sus scrofa* genes with one or alternative poly(A) sites. We analyzed the number of PAS sequences per annotated gene, and grouped them as having 1, 2, 3, 4, 5, or over 5 PAS sequences.

First, we compared the distribution of the 21,640 annotated protein-coding genes [33] and the 62,491 pA sites identified above on the 18 autosomes and sex chromosomes (Figure 5A). The number of pA sites was roughly correlated with the number of protein-coding genes on all the chromosomes except for chromosome 1 and X chromosome, both of which had significantly fewer pA sites, relative to the number of genes on the chromosome (Figure 5A). We also compared the distribution of the pA sites and the annotated gene in the chromosome, the results showed that 73.94% (46,204 out of 62,491) of the pA sites were located in 12,801 annotated protein coding genes, and 26.06% pA sites (16,287 out of 62,491) was located in non-annotated gene regions, respectively (Figure 5B,C).

Among the 46,204 pA site associated with 12,801 genes, most of them (72.84%) were found in 3′UTRs (Figure 5B). The intron and exons of these genes were ranked as the 2nd and 3rd pA site-harboring regions, which had 22.00% and 4.34% pA sites, respectively. We also found a small number of pA site (0.82%) located in the 5′UTRs of these genes (Figure 5B). Among the 22,868 genes/transcripts mapped with pA sites, except for the unknown genes, 76.69% (exactly 9817) of genes have two or more alternative pA sites (Figure 5C), lading to variable length of 3′ coding or UTR sequence. 

### 3.6. APA Gene Participates in Diversity Function and Pathway in Pigs

6111 APA genes with more than two PASs were selected from a total of 22,861 genes to perform the gene ontology (GO) and Kyoto Encyclopedia of Genes and Genomes (KEGG) enrichment analysis.

**Figure 6:**
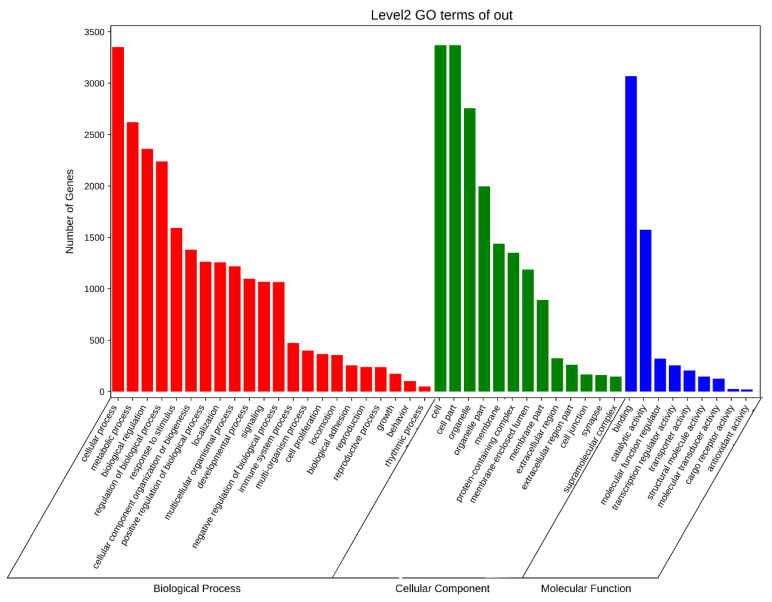
The GO enrichment analysis of genes in *Sus scrofa.* The genes whose PAS number are more than 2 (2 included) were selected to do enrichment tests in order to reveal the enrichment of gene ontology terms. Statistically significant molecular functions, biological processes, and pathways were identified. Each color block represents the gene amount in each tissue.

Gene ontology analysis revealed that different tissues cluster in distinct branches. Based on the gene ontology classification, differently expressed genes were characterized within three groups: biological processes, cellular components and molecular functions. The results showed that the APA gene mainly participates in cellular process, metabolic process and biological regulation process. In the cellular component category, the gene prefers to gather at cell, organelle and membrane. As for the molecular function, the APA gene is more related to binding and catalytic activity, which, respectively, has more than 3000 and 1500 genes involved (Figure 6).

**Figure 7:**
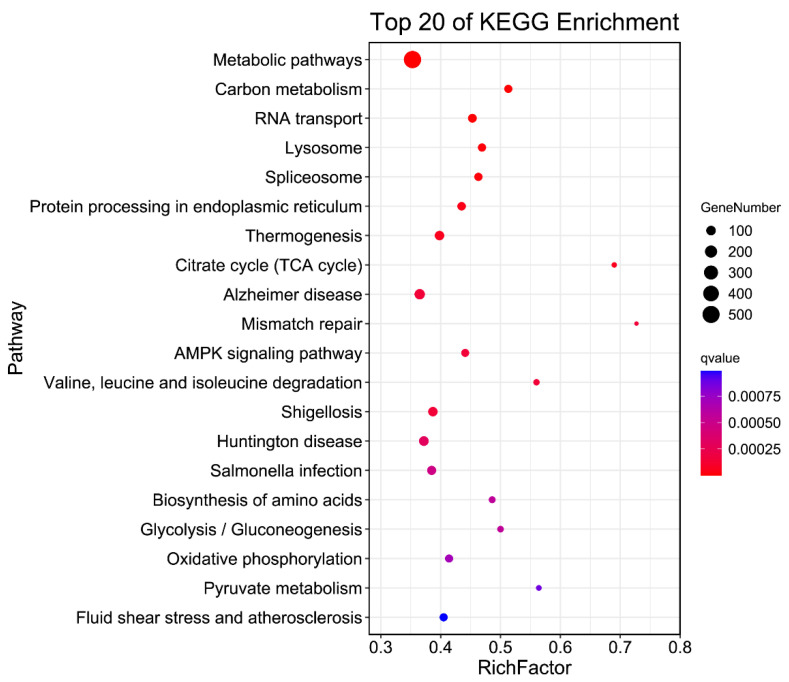
The Kegg enrichment analysis of genes in *Sus scrofa*. Scatter diagram of enrichment of genes with 2 or more than 2 PAS KEGG pathways. The *X*-axis shows the ratio of the number of annotated genes to the total pathway genes annotated, and the *Y*-axis shows the top 20 pathway names. The different color of dot indicates different value. The dot size indicates the gene number.

Visualizing the pathway enrichment analysis results of KEGG, we got a top 20 pathways gradient picture of the enrichment. The results that there are most genes enriching in the metabolic pathways. Furthermore, the carbon metabolism, RNA transport, lysosome, splicesome pathways were also highly enriched (Figure 7).

## 4. Discussion

More and more genomic and transcriptome data are produced as technologies are developed for high throughput sequencing technologies. With a comprehensive collection of poly(A) site data in *Sus scrofa*, we conducted the analysis of the feature of poly(A) sites, polyadenylation signals, and APA widely usage. Our results provide insight into RNA processing of polyadenylation and potential gene regulation by APA in *Sus scrofa*. We identified cleavage and polyadenylation sites in *Sus scrofa* as described previously [35] to perform the first analysis of PAS usage in livestock. Over 1 million individual tags and reads were mapped, and a total of 62,491 CSs were identified. Although the *Sus scrofa* genome is annotated, it did not distinguish different isoforms generated by APA. One important finding is the apparent widespread use of APA in *Sus scrofa*. 

Many strategies have been proposed to identity cleavage and polyadenylation sites [7], but special sequencing protocols are required. Prior research has produced a significant amount of transcriptome data and EST data, so we analyzed these data to obtain more information about polyadenylation. Tian’s study reported 29,823 poly(A) sites in human and 16,282 poly(A) sites in mouse. For comparison, we identified 62,491 poly(A) sites in *Sus scrofa*, a reasonable number of sites given the size of the genome. Our results suggest that many mRNAs in *Sus scrofa* can have different isoforms that only differ in the cleavage site location. The data suggest that APA regulation in the *Sus scrofa* is widely used to regulate gene expression. Given the regulatory role of polyadenylation in transcription, the recognition of polyadenylation signal (PAS) motif sequence becomes an important step. Although there have been methods predicting PAS successfully, the PAS identification still has room for improvement. In Yu’s study, they proposed a deep neural network-based computational method, called SANPolyA, for identifying PAS in human and mouse genomes [36]. In another study, a comprehensive and easily accessible APA site database for plants is presented [37]. What is more, after uniform analyzing, clustering, annotating and quantifying the poly(A) sites, constructed from publicly available human, mouse and worm 3′ end sequencing datasets, a consolidated atlas of polyadenylation sites named PolyASite 2.0 was generated [38]. 

APA can affect mRNA fate, and longer UTRs are not good for the gene expression because they offer an increased number of miRNA binding sites [39] and sites for RNA binding proteins. Common PAS variants were found with similar distributions as reported in human and fly [14]. The AAUAAA/AUUAAA and other PAS variants have similar PAS usage frequency, and these PAS were all recognized by the CPSF. This suggests that the polyadenylation process was conserved in different animals, although with some differences. Interestingly, for 10.64% of *Sus scrofa* transcripts, we did not find the previously reported PAS sequences upstream of cleavage sites [11]. The absence of these signals indicates alternative PAS sequences that have not been identified or the presence of an alternative mechanism. Compared to Beau and Tian’s research [13], our results have a lower frequency of unknown PAS, and a higher proportion of known PAS variants. However, this analysis may be affected by many factors. Since this analysis depends on RNA-seq data, the RNA-seq data quality is highly important for effective PAS analysis. Having a sufficient number of reads is also essential, and this analysis utilized a larger amount of data than the previous studies. At the same time, the sequence data is much richer in PAS sequences in human and mouse. 

There are important future directions of this research. Even though we identified 62,491 poly(A) sites, only 73.94% percent of these poly(A) sites were able to be mapped to genes. This is likely at least partially due to the incomplete annotation of the *Sus scrofa* genome data. This data may also include false poly(A) sites. To solve these problems, we need more transcriptome data information for different *Sus scrofa* tissues. In addition, with the sequencing technology development, more and more long-read sequencing technologies was used in the transcriptomes study. Long-read sequencing were more benefit for the polyadenylation research rely on its no product amplification and reads assembly [40]. Future polyadenylation research the new sequence technology data also should be considered.

The PAS data presented in this manuscript will be useful for *Sus scrofa* research by facilitating the improved annotation of the *Sus scrofa* genome. Furthermore, we expect these data will serve as a basis for comparative studies of polyadenylation between various organisms.

## 5. Conclusions

In summary, our study provided the systematic analysis of the feature of poly(A) sites, polyadenylation signals, and gene regulation by APA levels in *Sus scrofa* genome. Among the identified 62,491 pA sites, we analyzed sequence feature around the cleavage site and count the canonical hexamer. The analysis of polyadenylation signal usage in *Sus scrofa* would give the new insights to future investigations into the mechanisms of transcriptional APA regulation.

## Figures and Tables

**Table 1:** Summary of all mRNA transcripts retrieved from the EST and RNA-seq databases of *Sus scrofa.*

Data Types	EST	RNA-Seq
Raw reads	1,676,405	36,890,214,409
poly(A) tail-containing reads	105,103	352,764,416
poly(A) tail-containing reads uniquely mapped to the genome	69,602	10,938,823
Cleavage sites	14,845	60,277
Total	62,491	

**Table 2:** Frequency (%) of PAS motifs.

PAS Motif	Frequency (%)
AATAAA	50.96
ATTAAA	12.39
TATAAA	4.45
AGTAAA	3.93
AAGAAA	2.63
AATATA	2.25
AATACA	2.23
CATAAA	2.25
GATAAA	2.09
AATGAA	0.59
TTTAAA	1.15
ACTAAA	1.36
AATAGA	1.21
AAAAAG	1.89
None	10.64

## Data Availability

The data presented in this study are available on request from the corresponding author.

## Supplementary Materials

The following are available online at https://www.mdpi.com/article/10.3390/ani12020194/s1, Table S1: SRA accession number.
